# The Protective Effect of Repeated 1MeTIQ Administration on the Lactacystin-Induced Impairment of Dopamine Release and Decline in TH Level in the Rat Brain

**DOI:** 10.1007/s12640-018-9939-6

**Published:** 2018-08-20

**Authors:** Agnieszka Wąsik, Irena Romańska, Agnieszka Zelek-Molik, Irena Nalepa, Lucyna Antkiewicz-Michaluk

**Affiliations:** 1 Department of Neurochemistry, Institute of Pharmacology, Polish Academy of Sciences, Smętna 12, 31-343 Kraków, Poland; 2 Department of Brain Biochemistry, Institute of Pharmacology, Polish Academy of Sciences, Smętna 12, 31-343 Kraków, Poland

**Keywords:** Parkinson’s disease (PD), Ubiquitin-proteasome system (UPS), Lactacystin, 1-Methyl-1,2,3,4-tetrahydroisoquinoline (1MeTIQ), Microdialysis study

## Abstract

Parkinson’s disease (PD) is a neurodegenerative disorder of the central nervous system (CNS) caused by a progressive loss of nigrostriatal dopaminergic neurons. Dysfunction of the ubiquitin-proteasome system (UPS) plays an important role in the pathogenesis of PD. Intranigral administration of the UPS inhibitor lactacystin is used to obtain a valuable animal model for investigating putative neuroprotective treatments for PD. 1-Methyl-1,2,3,4-tetrahydroisoquinoline (1MeTIQ) is an endogenous amine that displays neuroprotective properties. This compound acts as a reversible monoamine oxidase (MAO) inhibitor and a natural free radical scavenger. In the present experiment, we investigated the effect of acute and chronic treatment with 1MeTIQ on locomotor activity and the release of dopamine as well as its metabolites in the striatum of unilaterally lactacystin-lesioned and sham-operated rats using in vivo microdialysis. Additionally, changes in the level of tyrosine hydroxylase (TH) in the substantia nigra were measured. Unilateral lactacystin injection into the substantia nigra caused significant impairment of dopamine release (approx. 45%) and a marked decline in the TH level. These effects were completely antagonized by multiple treatments with 1MeTIQ. The results obtained from the in vivo microdialysis study as well as from the ex vivo experiments suggest that multiple administration of 1MeTIQ protects dopaminergic neurons against the lactacystin-induced decline in TH concentration in the substantia nigra and prevents disturbances of dopamine release in the striatum. We have demonstrated that 1MeTIQ is capable of maintaining the physiological functions of the striatal dopamine neurons damaged by unilateral lactacystin lesion.

## Introduction

Parkinson’s disease (PD) is a neurodegenerative disorder of the central nervous system (CNS) caused by a progressive loss of nigrostriatal dopaminergic neurons. This degeneration leads to typical clinical symptoms, such as bradykinesia, tremor, and rigidity. Additionally, PD is characterized by the presence of insoluble proteinaceous inclusions known as Lewy bodies, consisting of a wide range of proteins (α-synuclein, ubiquitin, neurofilaments) (Spillantini et al. 1998). Experimental studies have provided evidence that the dysfunction of the ubiquitin-proteasome system (UPS) plays a vital role in the pathogenesis of PD (Martins-Bianco et al. 2012; McNaught and Jenner 2001). The UPS is a major multicatalytic proteinase complex that is responsible for the degradation of unwanted and misfolded proteins (Ciechanover 1998; Sherman and Goldberg 2001). Lactacystin, a selective proteasome inhibitor, has been used to model PD in animals (Fenteany and Schreiber 1998). In rodents, nigral administration of lactacystin produces a fast-onset PD-like phenotype, including α-synuclein elevation, tyrosine hydroxylase (TH)-positive cell loss, and behavioral deficits (Bentea et al. 2015; Lorenc-Koci et al. 2011; McNaught et al. 2002). Therefore, the lactacystin model can be valuable for investigating putative neuroprotective treatments for PD.

1-Methyl-1,2,3,4-tetrahydroisoquinoline (1MeTIQ) is an endogenous compound from the tetrahydroisoquinoline group that is present in the dopaminergic structures of the mammalian brain (Abe et al. 2005; Yamakawa and Ohta 1997, 1999; Yamakawa et al. 1999). When administered intraperitoneally, 1MeTIQ can easily passed through the blood-brain barrier and concentrates in the brain (Kikuchi et al. 1991). As previously demonstrated by our team, 1MeTIQ displays neuroprotective activity both in vitro (Antkiewicz-Michaluk et al. 2006) and in vivo in several animal models of PD (Antkiewicz-Michaluk et al. 2003, 2004, 2014; Wąsik et al. 2016a, b, 2017). Our earlier experimental studies determined that 1MeTIQ acts as a reversible MAO (monoamine oxidase) inhibitor and blocks the MAO-dependent oxidative pathway with the simultaneous activation of the COMT (catechol-O-methyltransferase)-dependent O-methylation catabolic pathway. As a result, 1MeTIQ inhibits free radical formation generated during dopamine-oxidation via the Fenton reaction and consequently exhibits essential antioxidant properties (Antkiewicz-Michaluk et al. 2006; Patsenka and Antkiewicz-Michaluk 2004; Singer and Ramsay 1995). These results revealed that 1MeTIQ exerts its effects independent of a direct interaction with biological structures, possesses intrinsic antioxidant properties, and acts as a natural scavenger of free radicals.

The purpose of the current research was to evaluate the effect of acute and chronic with 1MeTIQ on locomotor activity and the extraneuronal concentration of dopamine and its metabolites DOPAC (3,4-dihydroxyphenylacetic acid), 3-MT (3-methoxytyramine), and HVA (homovanillic acid) in the striatum of unilaterally lactacystin-lesioned and sham-operated rats using in vivo microdialysis. In addition, the effect of lactacystin lesion on the level of tyrosine hydroxylase (TH) in the substantia nigra was measured using Western blot.

## Materials and Methods

### Animals and Treatments

**Scheme 1:**
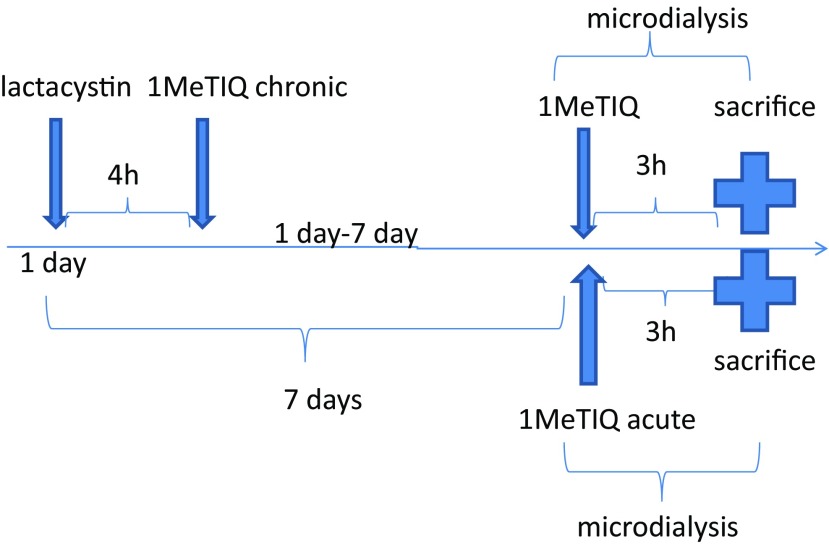
The substance administration schedule

All experiments were carried out on male Wistar rats with an initial body weight of 280–300 g. All animals had free access to standard laboratory feed and tap water and were kept at room temperature (22 °C) under an artificial light/dark cycle (12/12 h, light on at 7:00 h). After surgery, the rats used for the microdialysis study were individually housed for a couple of days. The experiments were carried out between 09:00 and 16:00 h. Control rats were treated with an appropriate solvent (0.9% NaCl). The rats were administered 1MeTIQ at a dose of 50 mg/kg intraperitoneally (i.p.) either acutely (7 days after lactacystin lesion) or as multiple doses (during seven consecutive days, 7 × 50 mg/kg; first injection given 4 h after lactacystin lesion) (Scheme 1).

All experimental procedures were carried out in accordance with the Guide for the Care and Use of Laboratory Animals issued by the National Institutes of Health and received approval from the Bioethics Commission as compliant with the Polish law. All experimental procedures were approved by the Local Bioethics Commission of the Institute of Pharmacology, the Polish Academy of Sciences in Kraków.

### Surgical Procedure

The rats were anesthetized with ketamine (75 mg/kg) and xylazine (10 mg/kg) and secured in a stereotaxic frame (Stoelting, USA). A stainless-steel needle was inserted unilaterally through a small hole in the skull, and the needle tip was placed in the right substantia nigra (SN) with the following stereotaxic coordinates: − 5.5 mm anterior/posterior (A/P) and − 2.0 mm lateral (L/M) from the bregma and − 7.8 mm ventral/dorsal (V/D) from the dura. The lactacystin solution was freshly prepared immediately prior to surgery. Lactacystin, at a dose of 5 μg (calculated as the free base) dissolved in a volume of 2 μl distilled water, was slowly infused into the right substantia nigra at a flow rate of 0.5 μl/min using a 10-μl Hamilton syringe. After termination of lactacystin infusion, the cannula was left in place for an additional 4 min for complete diffusion of the toxin and then slowly retracted. In this way, a unilateral lesion of the substantia nigra was made using the neurotoxin lactacystin. Sham-operated rats were treated in the same manner but received equivalent volumes of a vehicle instead of lactacystin. Implantation of the cannula was performed immediately after lactacystin administration during the same operation/anesthesia. Vertical microdialysis guide cannulas (Intracerebral Guide Cannula with Stylet; BAS Bioanalytical, USA) were implanted into the right striatum (STR) according to the following stereotaxic coordinates: + 1.0 mm A/P, − 2.5 mm L/M, and − 3.5 mm V/D from the bregma and dura (G. Paxinos and C.H. Watson).

### Drugs

Lactacystin (Sigma-Aldrich, USA) was obtained commercially. 1-Methyl-1,2,3,4-tetrahydroisoquinoline (1MeTIQ) was synthesized (according to Cannon and Webster 1958) at the Department of Drug Chemistry of the Institute of Pharmacology, the Polish Academy of Sciences in Kraków. The purity of the compounds was verified by measuring the melting point and assessing homogeneity in a chromatographic column. The compounds were dissolved in a 0.9% NaCl solution.

### Behavioral Study

#### Locomotor Activity

The locomotor activity and rearing were measured using actometers (Opto-Varimex activity monitors; Columbus Inst., USA) linked online to an IBM-PC compatible computer. Each cage (43 × 44 × 25 cm) was surrounded by a 15 × 15 array of photocell beams located 3 cm from the floor surface. Interruptions of these photocell beams were counted as a measure of horizontal locomotor activity defined as the distance traveled (in cm). Horizontal locomotor activity was recorded for 45 min (exploratory activity) and analyzed using the Auto-Track Software (Columbus Inst., USA).

1MeTIQ (50 mg/kg i.p.) was administered acutely or chronically. The control group was treated with saline. Immediately after 1MeTIQ injections, the animals were transferred to the experimental cages. Seven animals from each group were subjected to behavioral analysis.

### Biochemical Study

#### In Vivo Microdialysis

Seven days after surgery, microdialysis probes were inserted into the cannulas, and the striatum was perfused with an artificial cerebrospinal fluid (aCSF) consisting of 140 mM NaCl, 2.7 mM KCl, 1.2 mM CaCl_2_, 1 mM MgCl_2_, 0.3 mM NaH_2_PO_4_, and 1.7 mM Na_2_HPO_4_ (pH 7.4) at a flow rate of 1.5 μl/min using a microinfusion pump (Stoelting, Illinois USA). Samples were collected from freely moving rats at 20-min intervals after a 3-h washout period. Control (sham-operated) and lesioned (lactacystin) animals received saline injections, and dialysis samples were collected for 180 min. In the mixed groups, 1MeTIQ (50 mg/kg i.p.) was administered acutely (with the first administration 7 days after lactacystin lesion) or chronically (for seven consecutive days; with the first administration 4 h after lactacystin lesion). All dialysates were immediately frozen on dry ice (− 70 °C) until they were used for biochemical assay.

Levels of dopamine and its extraneuronal metabolite, 3-MT, were assayed in dialysates (20 μl) using HPLC with electrochemical detection as described below. The Dionex Ultimate 3000 chromatograph (Coulochem III, Germany) was equipped with C18 columns (Hypersil Gold; 150 mm × 3 μm). The mobile phase comprised 0.05 M citrate-phosphate buffer (pH 3.5), 0.1 mM EDTA, 1 mM sodium octyl sulfonate, and 3.5% methanol. The flow rate was maintained at 0.75 ml/min. Chromatographic data were processed using the Chromeleon Dionex computer software (Germany). Dopamine and its metabolites were quantified by chromatographic peak height and compared with standards run on the day of the analysis. At the end of the experiment, frozen brains were examined histologically for correct probe placement. Each group consisted of six animals.

### Immunoblotting

Protein was extracted through high-speed shaking in plastic tubes with stainless-steel beads in a tissue lyser with 100 μl ice-cold RIPA lysis buffer (Sigma, USA) containing a complete mini protease inhibitor (Roche Diagnostics, USA). After incubation for 30 min, the homogenates were centrifuged at 10,000*g* for 20 min at 4 °C. The resulting supernatants were collected and subjected to protein analysis using the Bicinchoninic Acid Assay Kit (Sigma, USA). Next, the standard Western blot procedure was conducted as previously described (Sun et al. 2013). Briefly, equal amounts of protein extracts (12 μg) were boiled in Laemmli buffer containing 5% β-mercaptoethanol for 5 min, separated through SDS-PAGE (4–15%) and transferred to nitrocellulose membranes. The membranes were blocked with 5% nonfat dry milk in Tris-buffered saline containing Tween-20 (TBST; pH = 7.6) for 1 h at room temperature and incubated overnight at 4 °C with a primary antibody against tyrosine hydroxylase (1:2000; Cell Signaling, USA). After three washes with the blocking solution, the membranes were incubated with the appropriate secondary antibodies for 1 h at room temperature, followed by three washes with TBST. Antibody binding was detected using an enhanced chemiluminescence kit (ECL Plus, Pierce, USA). Equal loading of protein per sample was further confirmed after probing with anti-β-actin antiserum (1:5000; Sigma, USA). All Western blot analyses were performed at least twice to confirm the results. The chemiluminescence signal was visualized using a Luminescent Image Analyzer Fuji-Las 4000 (Fuji, Japan). Immunoreactive bands were quantified using an image analyzer (ScienceLab, MultiGauge V3.0). Each group consisted of six animals.

### Statistical Analysis

The locomotor activity and immunoblotting results were analyzed by one-way analysis of variance (ANOVA) followed by Duncan’s post hoc test when appropriate. The microdialysis data were analyzed by one-way analysis of variance (ANOVA) for repeated measures, followed by Duncan’s post hoc test (if significant differences arose).

## Results

### Behavioral Tests

#### Locomotor Activity

**Fig. 1:**
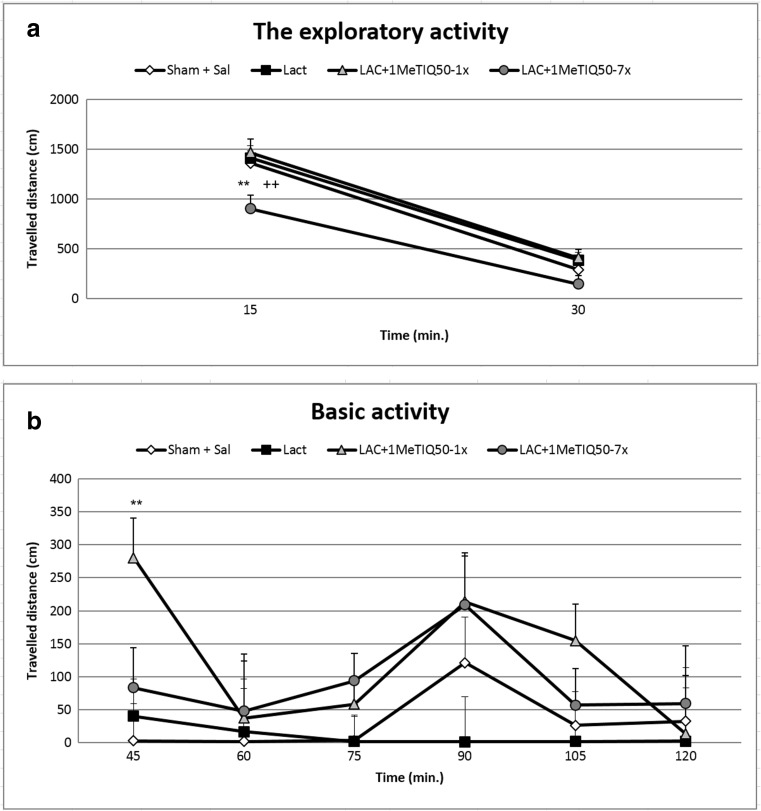
The effect of acute or chronic administration of 1MeTIQ and lactacystin-induced lesion on the exploratory (**a**) and basal (**b**) locomotor activity of rats. Sham-operated rats received a single injection of saline (control). Lactacystin was infused unilaterally into the right substantia nigra (5 μg) 7 days before the measurement of the locomotor activity. In the mixed group, 1MeTIQ (50 mg/kg i.p.) was administered acutely (on the day of the experiment) or chronically for seven consecutive days. Rats were placed into actometers immediately after the treatment. Movements were recorded for 120 min. The data are expressed as the means ± SEM (*n* = 6 animals). The data were analyzed with one-way ANOVA, followed by Duncan’s post hoc test. Statistical significance: *p < 0.05, **p < 0.01 vs. saline-treated group; ^+^*p <* 0.05 vs. lactacystin-lesioned group

The one-way ANOVA showed no statistical changes in the exploratory or basal locomotor activity of the rats in the lactacystin-lesioned group (Fig. 1a, b). In addition, reduced locomotor activity was observed in the first 15 min of the measurement in the lactacystin-lesioned group given 1MeTIQ (50 mg/kg) chronically (for 7 days) (*p* < 0.01) (Fig. 1a). Contrary to the results described above, acute administration of 1MeTIQ (50 mg/kg) combined with lactacystin lesion produced a significant increase (*p* < 0.05) in the basal activity of the rats at 45 min of the experiment (Fig. 1b).

### Biochemical Study

#### Dopamine Concentration in the Extracellular Space: In Vivo Microdialysis Study

##### The Effect of Unilateral Lactacystin Lesion

**Fig. 2:**
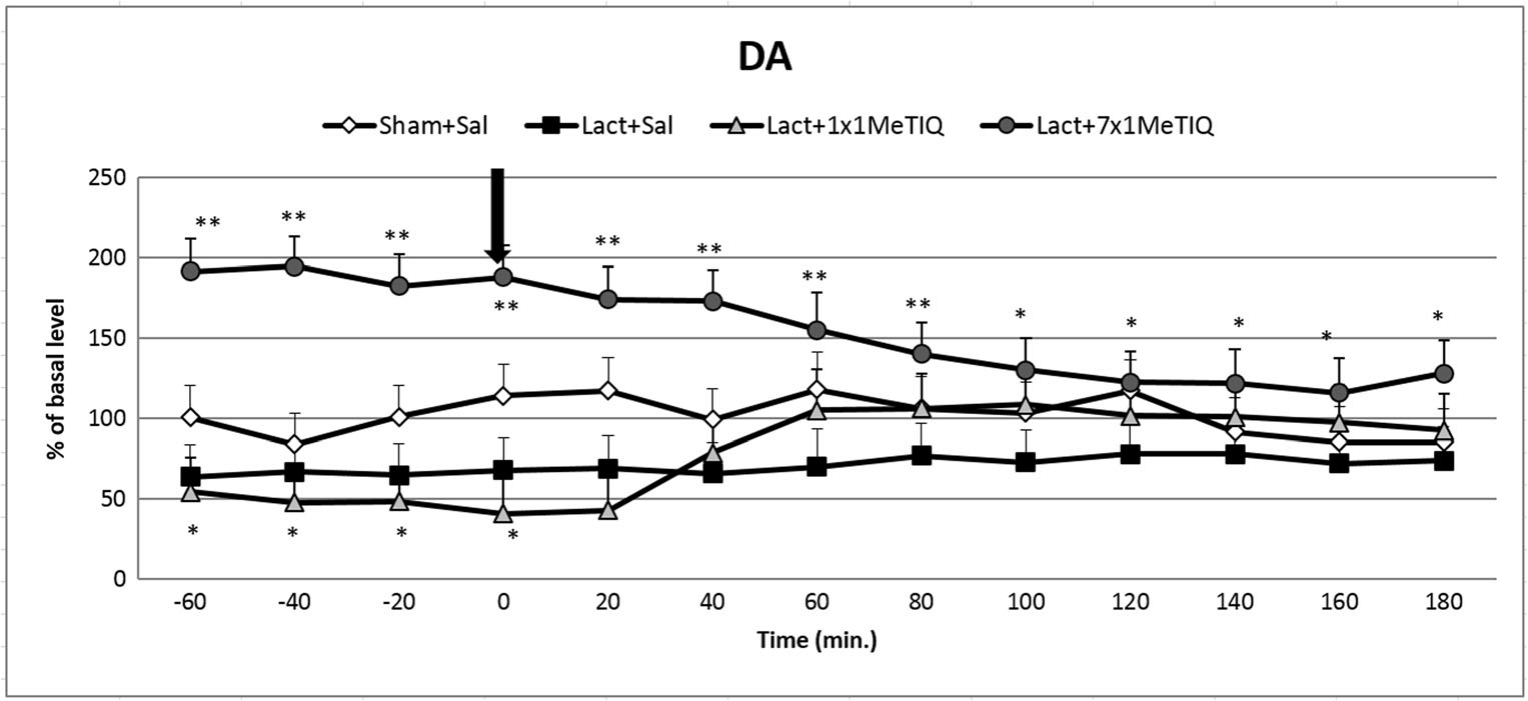
The effect of acute or chronic administration of 1MeTIQ on lactacystin-induced changes in dopamine release in the rat striatum. Sham-operated rats received a single injection of saline (control). Lactacystin was infused unilaterally into the right substantia nigra (5 μg) 7 days before the microdialysis study. In the mixed group, 1MeTIQ (50 mg/kg i.p.) was administered acutely (on the day of the experiment) or chronically for seven consecutive days. The dialysate was collected every 20 min. The concentration of dopamine (DA) was measured. The data are expressed as the means ± SEM (*n* = 6). Statistical significance: *p < 0.05, **p < 0.01 from the basal value (Duncan’s test)

The mean control basal extracellular concentration of dopamine in dialysates obtained from the striatum amounted to approximately 27.2 ± 2.6 (pg/20 μl). Statistical analysis demonstrated a significant reduction in the extracellular dopamine concentration (approx. 45% decrease, *F*[3,19] = 5.43; *p* < 0.01) in the basal samples (from − 60 to 0 min) 7 days after unilateral lactacystin lesion in comparison with that in the control group (saline) (Fig. 2). This toxic effect was observed until the end of the experiment.

##### The Impact of Acute or Chronic Administration of 1MeTIQ on Lactacystin-Induced Changes in Dopamine Release in the Rat Striatum

One-way ANOVA with repeated measures indicated a significant effect of 1MeTIQ treatment (*F*[3,19] = 5.43; *p* < 0.01). The post hoc test demonstrated that an acute dose of 1MeTIQ (50 mg/kg i.p.) administered 7 days after lactacystin inhibited the toxic effect of the lesion and restored the dopamine concentration up to the control level (from 60 to 180 min) (Fig. 2). Moreover, in the mixed group, repeated injections of 1MeTIQ (50 mg/kg i.p.) completely blocked the effect induced by lactacystin lesion, and the concentration of dopamine in basal samples was significantly elevated above that in the control (saline) group (approx. 200%) (Fig. 2).

### DOPAC Concentration in the Extracellular Space

#### The Effect of Unilateral Lactacystin Lesion

**Fig. 3:**
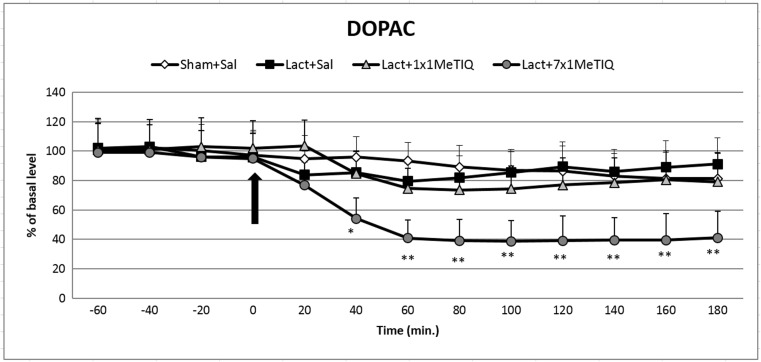
The effect of acute or chronic administration of 1MeTIQ on lactacystin-induced changes in DOPAC concentration in the rat striatum. Sham-operated rats received a single injection of saline (control). Lactacystin was infused unilaterally into the right substantia nigra (5 μg) 7 days before the microdialysis study. In the mixed group, 1MeTIQ (50 mg/kg i.p.) was administered acutely (on the day of the experiment) or chronically for seven consecutive days. The dialysate was collected every 20 min. The concentration of DOPAC was measured. The data are expressed as the means ± SEM (*n* = 6). Statistical significance: *p < 0.05, **p < 0.01 from the basal value (Duncan’s test)

Statistical analysis demonstrated that unilateral lactacystin lesion did not change the concentration of DOPAC (*F*[3,19] = 1.04; *N.S.*) in the rat striatum (Fig. 3).

#### The Impact of Acute or Chronic Administration of 1MeTIQ on Lactacystin-Induced Changes in the Concentration of DOPAC in the Rat Striatum

One-way ANOVA indicated that 1MeTIQ treatment produced no significant effect (*F*[3,19] = 1.04; *N.S.*) in the lactacystin-lesioned rats. However, the post hoc test demonstrated that in the combined group, chronic administration of 1MeTIQ induced a significant decrease in the DOPAC concentration (approx. 60%) in the lactacystin-lesioned rats (Fig. 3).

### 3-MT Concentration in the Extracellular Space

#### The Effect of Unilateral Lactacystin Lesion

**Fig. 4:**
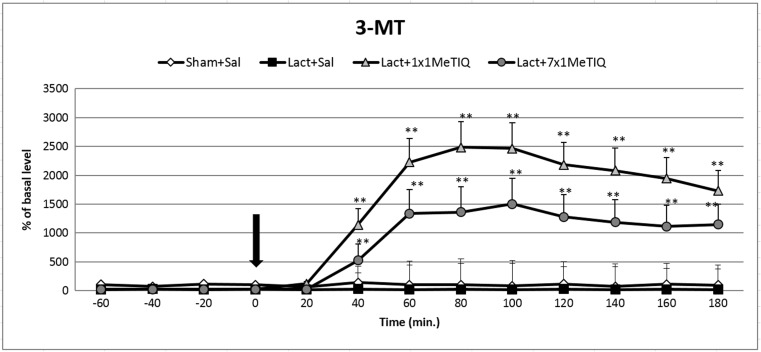
The effect of acute or chronic administration of 1MeTIQ on lactacystin-induced changes in the 3-MT concentration in the rat striatum. Sham-operated rats received a single injection of saline (control). Lactacystin was infused unilaterally into the right substantia nigra (5 μg) 7 days before the microdialysis study. In the mixed group, 1MeTIQ (50 mg/kg i.p.) was administered acutely (on the day of the experiment) or chronically for seven consecutive days. The dialysate was collected every 20 min. The concentration of 3-MT was measured. The data are expressed as the means ± SEM (*n* = 6). Statistical significance: *p < 0.05, **p < 0.01 from the basal value (Duncan’s test)

Analysis of variance indicated that the unilateral lactacystin-induced lesion of the substantia nigra significantly affected the 3-MT concentration in the rat striatum (*F*[3,18] = 6.16; *p* < 0.05) (Fig. 4). Duncan’s test showed that lactacystin lesion reduced the level of 3-MT by approximately 60% in comparison to the level in the control (saline) group throughout the entire measurement period (Fig. 4).

#### The Effect of Single or Chronic Administration of 1MeTIQ on Lactacystin-Induced Changes in the Concentration of 3-MT in the Rat Striatum

Statistical analysis indicated a significant effect of 1MeTIQ treatment (*F*[3,8] = 6.16; *p* < 0.05) in the lactacystin-lesioned rats. The post hoc test demonstrated that both acute and chronic administration of 1MeTIQ (50 mg/kg i.p.) produced a significant increase (approx. 2500 and 1500%, respectively) in the striatal concentration of 3-MT in the lactacystin-lesioned groups (*p* < 0.01) (Fig. 4).

### HVA Concentration in the Extracellular Space

#### The Effect of Unilateral Lactacystin Lesion

**Fig. 5:**
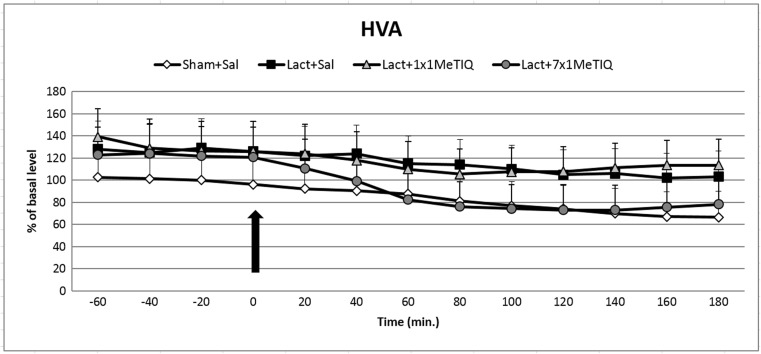
The effect of acute or chronic administration of 1MeTIQ on lactacystin-induced changes in the HVA concentration in the rat striatum. Sham-operated rats received a single injection of saline (control). Lactacystin was infused unilaterally into the right substantia nigra (5 μg) 7 days before the microdialysis study. In the mixed group, 1MeTIQ (50 mg/kg i.p.) was administered acutely (on the day of the experiment) or chronically for seven consecutive days. The dialysate was collected every 20 min. The concentration of HVA was measured. The data are expressed as the means ± SEM (*n* = 6). Statistical significance: *p < 0.05, **p < 0.01 from the basal value (Duncan’s test)

One-way ANOVA showed that unilateral lactacystin lesion did not change the concentration of the final dopamine metabolite, HVA (*F*[3,16] = 0.78; *N.S.*) (Fig. 5).

#### The Impact of Acute or Chronic Administration of 1MeTIQ on Lactacystin-Induced Changes in the Concentration of HVA in the Rat Striatum

Analysis of variance indicated that in the lactacystin-lesioned rats, the effect of treatment with 1MeTIQ was not significant (*F*[3,16] = 0.78; *N.S.*) (Fig. 5).

### The Rate of Dopamine Reuptake in the Extracellular Space

One-way ANOVA indicated a significant effect of treatment with 1MeTIQ (*F*[3,18] = 24.44; *p* < 0.01) in the lactacystin-lesioned rats. Duncan’s test demonstrated that both acute and chronic administration of 1MeTIQ (50 mg/kg i.p.) strongly elevated the rate of dopamine reuptake measured as an index of [3-MT]/[DOPAC] in the lactacystin-lesioned groups (Table 1).

**Table 1:** The effect of acute or chronic administration of 1MeTIQ on lactacystin-induced changes in the rate of dopamine reuptake ([3-MT]/[DOPAC] × 100) in the rat striatum

Treatment	[3-MT]/[DOPAC] × 100
Sham + Sal	112 ± 3
Lact + Sal	47 ± 10
Lact + 1MeTIQ 1×	2843 ± 526**^++^
Lact + 1MeTIQ 7×	2080 ± 220**^++^
*F*	*F*_(3/18)_ = 24.44 *P* < 0.01

Sham-operated rats received a single injection of saline (control). Lactacystin was infused unilaterally into the right substantia nigra (5 μg) 7 days before microdialysis study. In the mixed group, 1MeTIQ (50 mg/kg i.p.) was administered acutely (on the day of the experiment) or chronically for seven consecutive days. The dialysate was collected every 20 min. The results are expressed as the means ± SEM of five-six samples (*n* = 5–6 animals per group). Data were analyzed by means of one-way ANOVA followed by Duncan’s test. The rate of dopamine reuptake was assessed from the ratio of the extraneuronal dopamine metabolite concentration, 3-MT to the intraneuronal dopamine metabolite concentration, DOPAC and expressed as the index ([3-MT]/[DOPAC]) × 100. The indices were calculated using concentrations from individual tissue samples (*n* = 5–6). Statistical significance: **p < 0.01 versus saline group; ^++^*p* < 0.01 versus lactacystin group

### Tyrosine Hydroxylase Protein Level

**Fig. 6:**
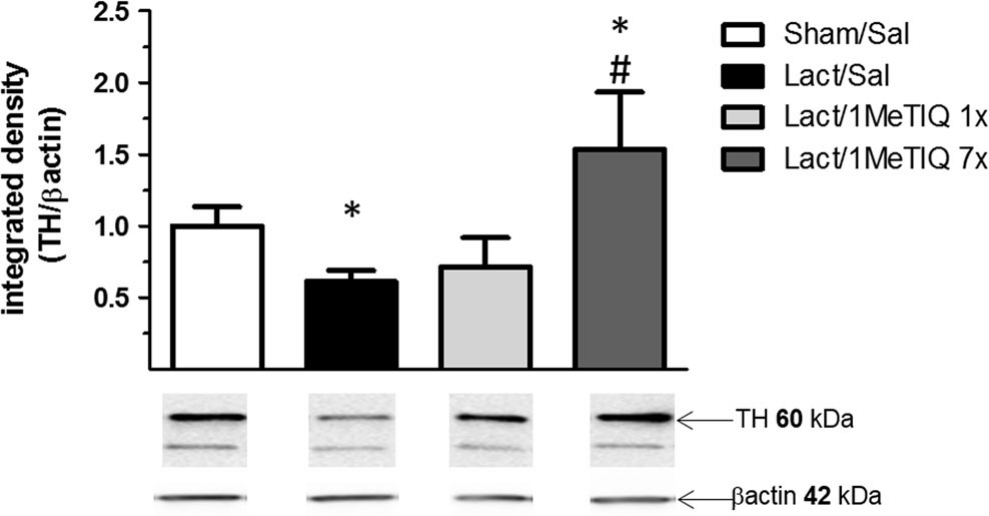
The effect of acute or chronic administration of 1MeTIQ on lactacystin-induced changes in the concentration of tyrosine hydroxylase (TH) in the substantia nigra of the rat. Sham-operated rats received a single injection of saline (control). Lactacystin was infused unilaterally into the right substantia nigra (5 μg) 7 days before the experiment. In the mixed groups, 1MeTIQ (50 mg/kg i.p.) was administered acutely (on the day of the experiment) or chronically for seven consecutive days. The right substantia nigra was dissected 3 h after the last 1MeTIQ administration. The data are expressed as the means ± SEM (*n* = 6 animals). The data were analyzed with one-way ANOVA, followed by Duncan’s post hoc test. Statistical significance: *p < 0.01 vs. saline-treated group; ^+^*p <* 0.05 vs. lactacystin-lesioned group

The data obtained from the ipsilateral side showed that lactacystin lesion produced a significant reduction (approx. 30%, *p* < 0.05) in the level of tyrosine hydroxylase in the substantia nigra (Fig. 6). In addition, repeated treatment with 1MeTIQ (50 mg/kg) completely prevented the toxic effect induced by lactacystin lesion, and in this group, the level of tyrosine hydroxylase was higher than that in the control group (Fig. 6).

## Discussion

Much of our current knowledge about the potential mechanisms underlying PD has been acquired as a result of conducting experiments using animal models. There are a number of different animal models of PD, among which intranigral administration of the proteasome inhibitor, lactacystin, represents one of the best. This model allows for the induction of nigrostriatal dopamine neurodegeneration and clinical manifestations such as motor disturbances as well as cytoplasmic accumulation of α-synuclein-positive aggregates in the dopaminergic neurons of the rat brain (Bentea et al. 2017; McNaught et al. 2002; Niu et al., 2009; Pienaar et al. 2015; Vernon et al. 2010). Recently, other authors showed a progressive and dose-dependent loss of dopaminergic neurons after lactacystin administration (Ahn and Jeon, 2006; Lorenc-Koci et al. 2011; Mackey et al. 2013; McNaught et al. 2002; Niu et al. 2009). Moreover, the death of dopaminergic neurons following lactacystin lesion was demonstrated to primarily be caused by apoptosis (Niu et al. 2009).

1MeTIQ is the member of the noncatechol 1,2,3,4-tetrahydroisoquinoline family with the least complicated structure and is present naturally in plants as well as the brain of humans, other primates, and rodents. The key finding of the present study was that chronic administration of this endogenous amine completely prevented the disturbances in dopamine release induced by unilateral lactacystin lesion. In our experiment, intranigral lactacystin lesion induced a significant decrease (approx. 50%) in dopamine release in the striatum of the rat brain (Fig. 2). Furthermore, the concentration of extraneuronal dopamine in the basal samples from the combined group (lactacystin and chronic 1MeTIQ) was approximately 200% above the concentration in the control group (sham + saline; Fig. 2). These results are in agreement with our previous study demonstrating that 1MeTIQ in naïve, control rats produced a slight (from 150 to 200%) elevation in the extraneuronal concentration of dopamine (Wąsik et al. 2016a). Such an effect may suggest that 1MeTIQ is capable of inhibiting dopamine reuptake. In fact, our earlier in vitro study revealed that 1MeTIQ in low micromolar concentrations displays activity as an inhibitor of the dopamine transporter (DAT) (Patsenka et al. 2004). In the present paper, we showed that an acute dose of 1MeTIQ administered on the seventh day after lactacystin lesion was able to increase the extraneuronal concentration of dopamine to the control level (Fig. 2). In turn, repeated doses of 1MeTIQ prevented dopaminergic neuronal damage and resulted in an additional increase in the amount of dopamine in the extracellular space. Notably, the first dose of 1MeTIQ was administered 4 h after lactacystin injection, and even under such circumstances, 1MeTIQ was still able to prevent lactacystin-induced neurotoxicity during the next 7 days. These results demonstrate the importance of an early initiation of neuroprotective therapy. However, unilateral lactacystin lesion did not change the level of DOPAC, but repeated 1MeTIQ injection in the combined group induced a significant (approx. 60%) reduction in this dopamine metabolite (Fig. 3). These effects are characteristic of the mechanism of action of 1MeTIQ. As is well known, 1MeTIQ acts as a reversible MAO_A_ and MAO_B_ inhibitor (Patsenka and Antkiewicz-Michaluk 2004). Therefore, the conversion of dopamine to DOPAC is severely inhibited, resulting in a decrease in the level of this metabolite. Considerable scientific evidence has revealed that blockade of dopamine oxidation triggers neuroprotective effects (Segura-Aquilar et al. 2014; Weinreb et al. 2011). Additionally, as shown in the present paper (Fig. 4), intranigral lactacystin lesion strongly influences the 3-MT concentration by decreasing its level (by approx. 60%) in the basal samples. Interestingly, both acute and chronic administration of 1MeTIQ completely reversed the toxic effect of lactacystin and produced a tremendous increase in the 3-MT level (2500 and 1500%, respectively) (Fig. 4). This effect of 1MeTIQ is related to its ability to potentiate COMT activity and provides an explanation for the specific mechanism of action of this compound (Antkiewicz-Michaluk et al. 2006). As previously established, intensification of the dopamine methylation pathway is a beneficial and safe catabolic process, and in contrast to oxidation, dopamine methylation does not result in the formation of free radicals.

In the present microdialysis study, we assessed the extracellular concentration of dopamine as well as its metabolites to assess the dynamics of dopamine release and the rate of its metabolism. Dopamine is metabolized by MAO_A_ and MAO_B_ into the intraneuronal metabolite DOPAC and by COMT into the extraneuronal metabolite 3-MT.

The final dopamine metabolite, HVA, is a product of the alternate activity of MAO and COMT. In the striatum, MAO is expressed in the outer membrane of mitochondria in the axon terminals of catecholamine neurons (Westlund et al. 1993), while COMT is primarily expressed in the membrane of glial and some postsynaptic cells (Kaakkola 2000). 3-MT is a major metabolite of dopamine released in the striatum, in contrast to DOPAC, which seems to originate from intraneuronal deamination of dopamine that has not been released. Notably, the ratio of 3-MT to DOPAC (3-MT**/**DOPAC) can be used as a useful index of reuptake inhibition (Karoum et al. 1994).

In the present investigation, we observed a strong increase in the 3-MT/DOPAC ratio in the 1MeTIQ group (Table 1). These results confirmed a significant inhibition of dopamine reuptake by 1MeTIQ.

Some authors have indicated that lactacystin lesion induces motor disturbances in rats (Konieczny et al. 2014; Mackey et al. 2013; Niu et al. 2009). In contrast, we did not observe changes in rat locomotor activity measured 7 days after unilateral lactacystin lesion (Fig. 1). Simultaneously, multiple administration of 1MeTIQ concomitant with lactacystin lesion decreased the exploratory activity of rats in the first 15 min of the experiment (Fig. 1). Such a short, transient decrease in locomotor activity is characteristic of the 1MeTIQ mechanism of action and is associated with the tremendous in the 3-MT concentration in the extracellular space. As demonstrated previously, 3-MT shows affinity for α1-adrenergic and D1 and D2 receptors as an antagonist; therefore, 3-MT may play an important role as an inhibitory regulator counteracting excessive stimulation of catecholaminergic neurons (Alachkar et al. 2010; Antkiewicz-Michaluk et al. 2008).

The data obtained from the immunoblotting study demonstrated that unilateral lactacystin lesion induced a significant decrease (approx. 30%) in the level of tyrosine hydroxylase in the substantia nigra measured on the ipsilateral side (Fig. 6). These results confirmed that lactacystin lesion caused dopaminergic cell death. There is evidence that the toxic effect of lactacystin is dose- and time-dependent (Lorenc-Koci et al. 2011; Mackey et al., 2013; Shulman et al. 2011). The same data demonstrated that application of different proteasome inhibitors leads to apoptotic cell death via activation of caspase-3 and -9 and mitochondrial cytochrome c release (Lee et al. 2005; Li et al. 2008; Nair et al. 2006). Moreover, as shown by Fornai et al. [2003], lactacystin leads to the formation of intracellular aggregates containing ubiquitin, α-synuclein, and parkin. In our present experiment, the toxic effects of lactacystin were rather weak and could be used as a model of the early phase of PD. In this case, 1MeTIQ completely prevented the toxic effect of lactacystin, and the level of tyrosine hydroxylase was elevated above that in the control group (Fig. 6). These results are in agreement with those in the in vivo microdialysis study, as in both experiments, repeated treatment with 1MeTIQ showed neuroprotective activity against the lactacystin-induced impairment of the function of dopamine neurons in the rat brain. Moreover, a neuroprotective effect of 1MeTIQ on TH-positive cells has been observed by other authors using the MPTP model. MPTP is converted into MPP+ by the enzyme MAO_B_ (Parrado et al. 2000). In this model, MPP+ ions accumulate in the mitochondria and bind to complex I, causing the inhibition of mitochondrial respiration (Ramsay et al., 1986).

The results presented in this paper obtained from the in vivo microdialysis study and ex vivo molecular research suggest that repeated administration of 1MeTIQ protects dopaminergic neurons against lactacystin-induced disturbances of dopamine release. In addition, this endogenous amine was able to maintain physiological functions of the striatal dopamine neurons damaged by unilateral lactacystin lesion.

## Abbreviations

aCSF: Artificial cerebrospinal fluid
A/P: Anterior/posterior
CNS: Central nervous system
COMT: Catechol-O-methyltransferase
DAT: Dopamine transporter
DOPAC: 3,4-Dihydroxyphenylacetic acid
HPLC: High-performance liquid chromatography
HVA: Homovanillic acid
L/M: Lateral to the midline
MAO: Monoamine oxidase
1MeTIQ: 1-Methyl-1,2,3,4-tetrahydroisoquinoline
MPP^+^: 1-Methyl-4-phenylpyridinium ion
3-MT: 3-Methoxytyramine
PD: Parkinson’s disease
TH: Tyrosine hydroxylase
UPS: Ubiquitin-proteasome system
V/D: Ventral/dorsal
